# U-Shaped Association Between Blood Pressure and Mortality Risk in ICU Patients With Atrial Fibrillation: The MIMIC-III Database

**DOI:** 10.3389/fcvm.2022.866260

**Published:** 2022-06-20

**Authors:** Ying Shao, Jinzhu Hu

**Affiliations:** ^1^Department of Clinical Medicine, Queen Mary College of Nanchang University, Nanchang, China; ^2^Department of Cardiovascular Medicine, The Second Affiliated Hospital of Nanchang University, Nanchang, China

**Keywords:** blood pressure, atrial fibrillation, smooth curve, mortality, intensive care unit

## Abstract

**Background:**

Existing evidence on the association between blood pressure (BP) and mortality risk in intensive care unit (ICU) patients with atrial fibrillation (AF) is scarce.

**Aim:**

This study aimed to assess the associations between blood pressure (BP) and risks of in-hospital and all-cause mortality in ICU patients with AF.

**Methods:**

A total of 2,345 records of patients with AF whose BP was monitored after admission to the ICU were obtained from the MIMIC-III database. Incidences were calculated for endpoints (hospital mortality, 7-day mortality, 30-day mortality, and 1-year mortality). We performed smooth curve and logistic regression analyses to evaluate the association between BP and the risk of each endpoint.

**Results:**

Smooth curve regression showed that systolic blood pressure (SBP), mean arterial pressure (MBP), and diastolic blood pressure (DBP) followed U-shaped curves with respect to endpoints (hospital mortality, 7-day mortality, 30-day mortality, and 1-year mortality). The incidence of these endpoints was lowest at 110/70/55 mm Hg. There was an increased risk of 1-year mortality observed with BP > 110/70/55 mm Hg (SBP, odds ratio [*OR*] = 1.008, 95% *CI* 1.001–1.015, *p* = 0.0022; MBP, *OR* = 1.010, 95% *CI* 1.005–1.016, *p* < 0.001) after adjusting for age, sex, and medical history. In contrast, an inverse association between BP and the risk of 1-year mortality was observed with BP ≤ 110/70/55 mm Hg (SBP, *OR* = 0.981, 95% *CI* 0.974–0.988, *p* < 0.001; MBP *OR* = 0.959, 95% *CI* 0.939–0.979, *p* < 0.001; and DBP, *OR* = 0.970, 95% *CI* 0.957–0.983, *p* < 0.001).

**Conclusions:**

We observed a U-shaped association between BP and in-hospital/all-cause mortality in ICU patients with AF. However, the underlying causes need to be investigated.

## Introduction

As a common cardiac arrhythmia, atrial fibrillation (AF) has increased considerably in prevalence in the general population aged ≥65 years (1). Evidence from previous studies has demonstrated that AF strongly contributes to an increased long-term risk of all-cause mortality. Existing studies suggest that demographic characteristics, such as advanced age and male sex; lifestyle factors, such as high body mass index (BMI) and low levels of physical exercise; and history of the disease, such as hypertension, myocardial infarction, valvular disease, heart failure, and diabetes mellitus, are all important factors contributing to AF (2–4). However, hypertension may be more important than other factors (2, 5) due to its high prevalence in the general population. Consequently, hypertension tends to be the most important target in the prevention of AF.

Blood pressure (BP) also has an important effect on mortality. Every 20/10 mm Hg increase in BP doubles cardiovascular risk in seniors with BP >115/75 mm Hg (6, 7). Previous studies have confirmed the strong association between BP and cardiovascular events. For instance, in certain individuals, such as patients with acute coronary syndrome or older adults, a J-shaped association between BP and adverse outcomes has been observed (8, 9). Low BP (<110/70 mm Hg) is related to increased incidence of negative outcomes, with mortality risk lowest at BP values ranging from (130 to 140)/(80 to 90) mm Hg (8). Similar results have also been demonstrated in individuals with stroke and chronic coronary artery disease (CAD) (10–13). However, few studies exist on the association between BP and mortality in specific individuals with AF. Only one study focused only on patients with AF, reporting a U-shaped association of BP with all-cause mortality. Their results showed that the incidence of all-cause mortality was lowest at 140/78 mm Hg (14). These correlative differences may have been due to differences among participants in various demographic characteristics, lifestyles, comorbidities, and different statistical methods.

Patients in the intensive care unit (ICU), as a special department, have high mortality risk, of which patients with AF account for a certain proportion. Reducing the mortality of ICU patients with AF has always been a major clinical objective. However, no study has focused on the association between BP and mortality risk and the optimal BP target in ICU patients with AF (14). Considering the loss of atrial contractility, the optimal value of BP in patients with AF may differ from that in the general population, which would be of great clinical significance for defining thresholds of BP below which adverse events may increase or decline in frequency. Therefore, by using records of patients obtained from the MIMIC-III database, we investigated whether a strong association exists between BP and mortality in ICU patients with AF. Our main objective was to investigate the nonlinear association between BP and mortality (hospital mortality, 7-day mortality, 30-day mortality, and 1-year mortality) in a large cohort of patients with AF and determine the optimal BP at the lowest mortality. Furthermore, we attempted to evaluate the possible effects of age, sex, comorbidity, and medical treatment on the association of BP with mortality, and these confounding factors may be important moderators that few have previously taken into account.

## Materials and Methods

The data used in the present study were obtained from the MIMIC-III database (15). Briefly, the MIMIC-III database contains information on 46,520 patients admitted to the Beth Israel Deaconess Medical Center (BIDMC) from 2001 to 2012 (15). The establishment of this freely available database was approved by the Institutional Review Boards (IRBs) of the Massachusetts Institute of Technology (MIT) and BIDMC. The database includes demographic data, laboratory tests, fluid balance data, vital status and blood gas analysis data, discharge summaries, electrocardiography, imaging examinations, and diagnostic information. We included ICU patients diagnosed with AF using diagnosis codes from the International Classification of Diseases, Ninth Revision (ICD-9), and a total of 2,345 patients were considered eligible for inclusion in this study after excluding patients with the absence of important variables. The study was conducted in accordance with the Declaration of Helsinki. This was consistent with the Strengthening the Reporting of Observational Studies in Epidemiology (STROBE) statement (16).

### BP and Mortality

The BP was measured and recorded when entering the ICU, and the initial BP record values were further used for analysis in this study. The endpoints of the study were defined as hospital mortality, 7-day mortality, 30-day mortality, and 1-year mortality after the date of ICU admission. Hospital mortality was defined as death during hospitalization in the ICU. Furthermore, the 7-day mortality, 30-day mortality, and 1-year mortality were defined based on the time from the discharge date to the date of death.

### Confounding Variables

A large amount of admission information was collected for each patient from MIMIC-III by the Structured Query Language, such as demographic data (age and sex), laboratory results [white blood cell count (WBC), red blood cell count (RBC), platelet count (PLC), hemoglobin, serum creatinine, and blood urea nitrogen], medication records [β receptor blockers (βRBs), statins, nitrates, warfarin, and heparin], and clinical comorbidities [hypertension, chronic heart failure (CHF), valvular disease, chronic kidney disease (CKD), stroke, diabetes, chronic bronchitis, depression, and malignancy].

### Statistical Analysis

All statistical analyses in our study were conducted using SPSS 26.0 and EmpowerStats 3.0. Categorical data are presented as percentages, while continuous data are presented as the median (interquartile range, IQR). First, a smooth curve analysis was performed to determine the relationships between BP (systolic blood pressure [SBP], diastolic blood pressure [DBP], and mean arterial pressure [MBP]) and endpoints (hospital mortality, 7-day mortality, 30-day mortality, and 1-year mortality) and to further define the optimal value of BP with the lowest risk of mortality. According to the BP threshold, restrictive logistic regression models were then applied to determine whether BP was independently associated with endpoints (hospital mortality, 7-day mortality, 30-day mortality, and 1-year mortality) after adjusting for potential confounders. The crude model had no adjustment. Model 1 was adjusted for age and gender. Model 2 was adjusted for Model 1 plus CHF, valvular disease, and stroke. Model 3 was adjusted for Model 2 plus CKD, chronic bronchitis, depression, diabetes, and malignancy. Furthermore, interaction analysis was conducted to determine the impacts of belonging in various subgroups, classified by age, sex, CHF, valvular disease, hypertension, and medication (βRBs, statins, nitrates, warfarin, and heparin).

## Results

### Clinical Characteristics of Patients With AF in the ICU

**Figure 1:**
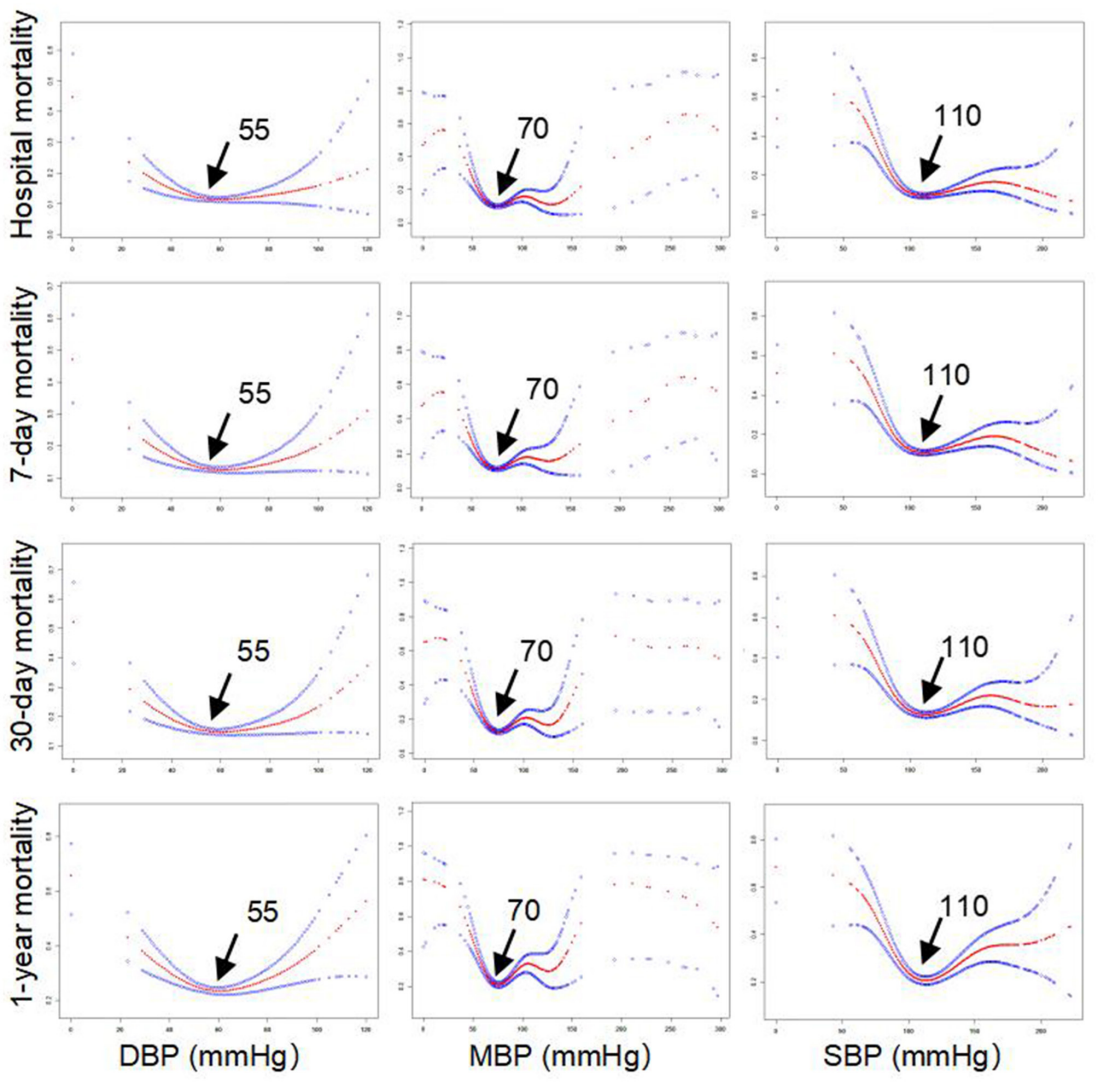
Smooth curve analysis of the association between blood pressure (BP) and mortality.

The clinical characteristics of these included patients with AF are presented in Table 1. Their median age was 73.6 years, and the number of men was 1,497 (63.8%). The median levels of SBP, MBP, and DBP were 114, 77, and 58 mm Hg, respectively. The incidence of hospital mortality, 7-day mortality, 30-day mortality, and 1-year mortality was 294 (12.54%), 326 (13.90%), 381 (16.25%), and 610 (26.01%), respectively. Other clinical information, such as comorbidities, medication, and blood biomarkers in the ICU, is also described in Table 1. Importantly, smooth curve analysis showed approximate U-shaped relations of SBP, MBP, and DBP with mortality (hospital mortality, 7-day mortality, 30-day mortality, and 1-year mortality), as shown in Figure 1. The BP levels with the lowest mortality risk, including those for SBP, MBP, and DBP, in these patients with AF were 110, 70, and 55 mm Hg, respectively.

**Table 1:** Clinical characteristics of ICU patients with AF.

**Variables**	**All *n =* 2,345**
	Median (interquartile range) or n (%)
Age	73.60 (65.43–79.66)
Gender (male)	1,495 (63.75%)
DBP (mmHg)	58.00 (50.00–66.00)
Max	82.00 (72.00–97.00)
Min	38.00 (30.00–44.00)
MBP (mmHg)	77.00 (69.00-88.00)
Max	111.00 (98.00–139.00)
Min	52.00 (45.00–58.00)
SBP (mmHg)	114.00 (102.00–129.00)
Max	159.00 (144.00–179.00)
Min	77.00 (61.00–87.00)
**Co-morbidity**	
Hypertension	1,206 (51.43%)
CHF	874 (37.27%)
Valvular disease	904 (38.55%)
Stroke	61 (2.60%)
Diabetes	39 (1.66%)
CKD	126 (5.37%)
Chronic bronchitis	37 (1.58%)
Depression	42 (1.79%)
Malignant	119 (5.07%)
**Medication**	
βRBs	1,636 (69.77%)
Statins	878 (37.44%)
Nitrates	355 (15.14%)
Warfarin	108 (4.61%)
Heparin	695 (29.64%)
**Blood biomarkers**	
RBC (m/uL)	3.33 (2.92–3.76)
PLC (K/uL)	158.00 (118.00–210.00)
WBC (K/uL)	12.00 (9.00–15.70)
Hemoglobin (g/dL)	10.00 (8.70–11.60)
Creatinine (mg/dL)	0.90 (0.70–1.20)
Urea nitrogen (mg/dL)	18.00 (14.00–27.00)
ICU stay (hour)	86.00 (49.00–185.00)
Hospital mortality	294 (12.54%)
7-day mortality	326 (13.90%)
30-day mortality	381 (16.25%)
1-year mortality	610 (26.01%)

*AF, atrial fibrillation; ICU, intensive care unit; DBP, diastolic blood pressure; MBP, mean blood pressure; SBP, systolic blood pressure; CHF, chronic heart failure; CKD, chronic kidney disease; βRB, β receptor blockers; RBC, red blood cell; PLC, platelet count; WBC, white blood cell count*.

### Multivariable Analysis Suggested a Significant Association of DBP With Mortality Stratified by the DBP Value With the Lowest Mortality Risk (55 mm Hg)

Based on the lowest of the BP thresholds reported above, stratified analysis was performed to evaluate the associations of DBP with mortality in our study. As shown in Table 2, increased DBP levels were associated with reduced risks of hospital mortality (odds ration [*OR*] = 0.961, 95% *CI* 0.949–0.973, *p* < 0.001, crude model), 7-day mortality (*OR* = 0.962, 95% *CI* 0.950–0.974, *p* < 0.001, crude model), 30-day mortality (*OR* = 0.963, 95% *CI* 0.951–0.975, *p* < 0.001, crude model), and 1-year mortality (*OR* = 0.965, 95% *CI* 0.953–0.976, *p* < 0.001, crude model) in AF patients with DBP ≤ 55 mm Hg. However, in patients with DBP > 55 mm Hg, increased DBP levels were only associated with the increased risk of 1-year mortality (*OR* = 1.012, 95% *CI* 1.001–1.023, *p* = 0.037, crude model) and not with those of hospital mortality (*OR* = 1.006, 95% *CI* 0.993–1.019, *p* = 0.376, crude model), 7-day mortality (*OR* = 1.008, 95% *CI* 0.996–1.020, *p* = 0.214 crude model), or 30-day mortality (*OR* = 1.007, 95% *CI* 0.996–1.019, *p* = 0.214, crude model). Importantly, the interactions (all values of *p* < 0.001) of hospital mortality, 7-day mortality, 30-day mortality, and 1-year mortality with the BP threshold (separating patients into a group with DBP ≤ 55 mm Hg and a group with DBP > 55 mm Hg) were significant. Furthermore, after adjusting for confounding factors, such as age, sex, CHF, valvular disease, stroke, CKD, chronic bronchitis, depression, diabetes, and malignancy, these independent associations in Model 3 were only slightly changed, and significant interactions for hospital mortality, 7-day mortality, 30-day mortality, and 1-year mortality still existed.

**Table 2:** Multiple logistic regression analysis for relationship between DBP and mortality risk.

**Variables**	**Hospital mortality**				**7-day mortality**				**30-day mortality**				**1-year mortality**			
	**OR**	**95% CI**	***P***	***P#***	**OR**	**95% CI**	***P***	***P#***	**OR**	**95% CI**	***P***	***P#***	**OR**	**95% CI**	***P***	***P#***
**Crude Model**																
DBP ≤ 55 mmHg	0.961	0.949–0.973	<0.001	<0.001	0.962	0.950–0.974	<0.001	<0.001	0.963	0.951–0.974	<0.001	<0.001	0.965	0.953–0.976	<0.001	<0.001
DBP>55mmHg	1.006	0.993–1.019	0.376		1.008	0.996–1.020	0.214		1.007	0.996–1.019	0.214		1.012	1.001–1.023	0.037	
**Model 1**																
DBP ≤ 55mmHg	0.960	0.948–0.972	<0.001	<0.001	0.962	0.950–0.974	<0.001	<0.001	0.962	0.950–0.974	<0.001	<0.001	0.963	0.951–0.975	<0.001	<0.001
DBP>55mmHg	1.006	0.994–1.019	0.347		1.008	0.996–1.020	0.199		1.008	0.996–1.020	0.199		1.014	1.002–1.026	0.026	
**Model 2**																
DBP ≤ 55mmHg	0.963	0.951–0.975	<0.001	<0.001	0.964	0.952–0.977	<0.001	<0.001	0.965	0.953–0.978	<0.001	<0.001	0.967	0.955–0.979	<0.001	<0.001
DBP>55mmHg	1.000	0.984–1.016	0.969		1.002	0.988–1.017	0.755		1.002	0.988–1.016	0.800		1.008	0.997–1.019	0.168	
**Model 3**																
DBP ≤ 55mmHg	0.965	0.953–0.978	<0.001	<0.001	0.967	0.955–0.980	<0.001	<0.001	0.968	0.955–0.980	<0.001	<0.001	0.970	0.957–0.983	<0.001	<0.001
DBP>55mmHg	1.001	0.986–1.017	0.869		1.004	0.990–1.018	0.587		1.003	0.989–1.017	0.660		1.009	0.998–1.020	0.113	

*Crude Model: No adjustment*.

*Model 1:Adjusted for age and gender*.

*Model 2:Adjusted for age, gender, CHF, valvular disease and stroke*.

*Model 3:Adjusted for age, gender, CHF, valvular disease, stroke, CKD, chronic bronchitis, depression, diabetes and malignant*.

*P#: P-value for interaction*.

*DBP, diastolic blood pressure; CHF, chronic heart failure; CKD, chronic kidney disease*.

### Multivariable Analysis Suggested a Significant Association of MBP With Mortality Stratified by the MBP Value With the Lowest Mortality Risk (70 mm Hg)

As shown in Table 3, higher MBP levels were related to reduced risks of hospital mortality (*OR* = 0.955, 95% *CI* 0.936–0.973, *p* < 0.001, crude model), 7-day mortality (*OR* = 0.956, 95% *CI* 0.938–0.975, *p* <0.001, crude model), 30-day mortality (*OR* = 0.951, 95% *CI* 0.932–0.970, *p* < 0.001, crude model), and 1-year mortality (*OR* = 0.953, 95% *CI* 0.934–0.972, *p* < 0.001, crude model) in AF patients with MBP ≤ 70 mm Hg. However, in patients with MBP > 70 mm Hg, higher MBP levels were associated with increased risks of hospital mortality (*OR* = 1.012, 95% *CI* 1.007–1.018, *p* < 0.001, crude model), 7-day mortality (*OR* = 1.012, 95% *CI* 1.007–1.018, *p* < 0.001, crude model), 30-day mortality (*OR* = 1.013, 95% *CI* 1.008–1.019, *p* < 0.001, crude model), and 1-year mortality (*OR* = 1.013, 95% *CI* 1.008–1.019, *p* < 0.001, crude model). Similarly, these independent associations in Model 3 were changed only slightly, and significant interactions with hospital mortality, 7-day mortality, 30-day mortality, and 1-year mortality still existed after adjusting for confounding factors, such as age, sex, CHF, valvular disease, stroke, CKD, chronic bronchitis, depression, diabetes, and malignancy.

**Table 3:** Multiple logistic regression analysis for relationship between MBP and mortality risk.

**Variables**	**Hospital mortality**				**7-day mortality**				**30-day mortality**				**1-year mortality**			
	**OR**	**95% CI**	***P***	***P#***	**OR**	**95% CI**	***P***	***P#***	**OR**	**95% CI**	***P***	***P#***	**OR**	**95% CI**	***P***	***P#***
**Crude Model**																
MBP ≤ 70mmHg	0.955	0.936–0.973	<0.001	<0.001	0.956	0.938–0.975	<0.001	<0.001	0.951	0.932–0.970	<0.001	<0.001	0.953	0.934–0.972	<0.001	<0.001
MBP>70mmHg	1.012	1.007–1.018	<0.001		1.012	1.007–1.018	<0.001		1.013	1.008–1.019	<0.001		1.013	1.008–1.019	<0.001	
**Model 1**																
MBP ≤ 70mmHg	0.954	0.936–0.973	<0.001	<0.001	0.956	0.938–0.974	<0.001	<0.001	0.950	0.931–0.969	<0.001	<0.001	0.952	0.933–0.971	<0.001	<0.001
MBP>70mmHg	1.012	1.007–1.018	<0.001		1.013	1.007–1.018	<0.001		1.013	1.008–1.019	<0.001		1.014	1.008–1.019	<0.001	
**Model 2**																
MBP ≤ 70mmHg	0.956	0.937–0.975	<0.001	<0.001	0.958	0.940–0.977	<0.001	<0.001	0.953	0.934–0.972	<0.001	<0.001	0.955	0.935–0.975	<0.001	<0.001
MBP>70mmHg	1.010	1.004–1.015	<0.001		1.010	1.004–1.015	<0.001		1.010	1.004–1.016	<0.001		1.010	1.005–1.016	<0.001	
**Model 3**																
MBP ≤ 70mmHg	0.960	0.941–0.979	<0.001	<0.001	0.962	0.943–0.981	<0.001	<0.001	0.956	0.936–0.975	<0.001	<0.001	0.959	0.939–0.979	<0.001	<0.001
MBP>70mmHg	1.010	1.004–1.016	<0.001		1.010	1.004–1.016	<0.001		1.010	1.005–1.016	<0.001		1.010	1.005–1.016	<0.001	

*Crude Model: No adjustment*.

*Model 1:Adjusted for age and gender*.

*Model 2:Adjusted for age, gender, CHF, valvular disease and stroke*.

*Model 3:Adjusted for age, gender, CHF, valvular disease, stroke, CKD, chronic bronchitis, depression, diabetes and malignant*.

*P#: P-value for interaction*.

*MBP, mean blood pressure; CHF, chronic heart failure; CKD, chronic kidney disease*.

### Multivariable Analysis Suggested a Significant Association of MBP With Mortality Stratified by the SBP Value With the Lowest Mortality Risk (110 mm Hg)

As shown in Table 4, our results suggested that increased SBP levels contributed to lower risks of hospital mortality (*OR* = 0.977, 95% *CI* 0.971–0.983, *p* < 0.001, crude model), 7-day mortality (*OR* = 0.978, 95% *CI* 0.971–0.984, *p* < 0.001 crude model), 30-day mortality (*OR* = 0.978, 95% *CI* 0.972–0.984, *p* < 0.001, crude model), and 1-year mortality (*OR* = 0.978, 95% *CI* 0.971–0.984, *p* < 0.001, crude model) in AF patients with SBP ≤ 110 mm Hg. However, in patients with SBP > 110 mm Hg, increased SBP levels only contributed to increased risks of 30-day mortality (*OR* = 1.009, 95% *CI* 1.002–1.017, *p* = 0.013, crude model), and 1-year mortality (*OR* = 1.013, 95% *CI* 1.007–1.020, *p* < 0.001, crude model) but not that of hospital mortality (*OR* = 1.008, 95% *CI* 0.009–1.016, *p* = 0.079, crude model), and 7-day mortality (*OR* = 1.008, 95% *CI* 1.000–1.016, *p* = 0.051, crude model). Furthermore, after adjustment for confounding factors, such as age, sex, CHF, valvular disease, stroke, CKD, chronic bronchitis, depression, diabetes, and malignancy, the independent associations in Model 3 remained significant, and the values of *p* of the interactions with hospital mortality, 7-day mortality, 30-day mortality, and 1-year mortality were < 0.001.

**Table 4:** Multiple logistic regression analysis for relationship between SBP and mortality risk.

**Variables**	**Hospital mortality**				**7-day mortality**				**30-day mortality**				**1-year mortality**			
	**OR**	**95% CI**	***P***	***P#***	**OR**	**95% CI**	***P***	***P#***	**OR**	**95% CI**	***P*** **Value**	***P#***	**OR**	**95% CI**	***P*** **Value**	***P#***
**Crude Model**																
SBP ≤ 110mmHg	0.977	0.971–0.983	<0.001	<0.001	0.978	0.971–0.984	<0.001	<0.001	0.978	0.972–0.984	<0.001	<0.001	0.978	0.971–0.984	<0.001	<0.001
SBP>110mmHg	1.008	0.999–1.016	0.079		1.008	1.000–1.016	0.054		1.009	1.002–1.017	0.013		1.013	1.007–1.020	<0.001	
**Model 1**																
SBP ≤ 110mmHg	0.977	0.971–0.983	<0.001	<0.001	0.977	0.971–0.983	<0.001	<0.001	0.978	0.971–0.984	<0.001	<0.001	0.977	0.970–0.983	<0.001	<0.001
SBP>110mmHg	1.006	0.998–1.015	0.163		1.006	0.998–1.015	0.124		1.008	1.000–1.016	0.040		1.012	1.006–1.019	<0.001	
**Model 2**																
SBP ≤ 110mmHg	0.979	0.973–0.985	<0.001	<0.001	0.979	0.973–0.985	<0.001	<0.001	0.980	0.973–0.986	<0.001	<0.001	0.979	0.972–0.986	<0.001	<0.001
SBP>110mmHg	1.002	0.993–1.011	0.738		1.002	0.993–1.011	0.643		1.004	0.996–1.012	0.376		1.009	1.002–1.016	0.009	
**Model 3**																
SBP ≤ 110mmHg	0.980	0.974–0.986	<0.001	<0.001	0.980	0.974–0.987	<0.001	<0.001	0.981	0.974–0.987	<0.001	<0.001	0.981	0.974–0.988	<0.001	<0.001
SBP>110mmHg	1.001	0.992–1.010	0.805		1.002	0.993–1.010	0.702		1.003	0.995–1.011	0.441		1.008	1.001–1.015	0.022	

***Crude Model:** No adjustment*.

***Model 1:**Adjusted for age and gender*.

***Model 2:**Adjusted for age, gender, CHF, valvular disease and stroke*.

***Model 3:**Adjusted for age, gender, CHF, valvular disease, stroke, CKD, chronic bronchitis, depression, diabetes and malignant*.

*P#: P-value for interaction*.

*SBP, systolic blood pressure; CHF, chronic heart failure; CKD, chronic kidney disease*.

### Analysis of Correlations Between BP and Mortality Stratified by Comorbidities and Medication

Interestingly, as shown in Table 5, in patients with MBP > 70 mm Hg, CHF (*p* = 0.026), nitrates (*p* < 0.001), and heparin (*p* = 0.021) modified the association between MBP and 1-year mortality. In patients with SBP > 110 mm Hg, nitrates modified the association between SBP and 1-year mortality (*p* = 0.019). Furthermore, hypertension (*p* = 0.002) and heparin (*p* < 0.001) modified the association between DBP and mortality in patients with DBP ≤ 55 mm Hg (Table 6). CHF (*p* = 0.046) and hypertension (*p* = 0.025) modified the association between MBP and mortality in patients with MBP ≤ 70 mm Hg, respectively, as well as in patients with SBP ≤ 110 mm Hg.

**Table 5:** Multiple logistic regression analysis for relationship between BP (>55/70/110 mmHg) and mortality by stratified analysis.

**Variables**	**Hospital mortality**				**7-day mortality**				**30-day mortality**				**1-year mortality**			
	**OR**	**95% CI**	***P***	***P#***	**OR**	**95% CI**	***P***	***P#***	**OR**	**95% CI**	***P***	***P#***	**OR**	**95% CI**	***P***	***P#***
**DBP**																
CHF	0.983	0.958–1.009	0.208	0.225	0.992	0.968–1.017	0.547	0.449	0.994	0.972–1.017	0.624	0.331	1.002	0.982–1.023	0.835	0.570
NO CHF	1.008	0.993–1.024	0.306		1.008	0.993–1.024	0.314		1.008	0.993–1.023	0.322		1.011	0.997–1.024	0.124	
Valvular disease	0.992	0.947–1.039	0.742	0.615	0.995	0.954–1.040	0.807	0.496	0.992	0.957–1.029	0.678	0.350	1.000	0.980–1.021	0.998	0.122
NO valvular disease	1.003	0.984–1.022	0.769		1.007	0.989–1.026	0.421		1.008	0.990–1.025	0.392		1.017	1.001–1.034	0.033	
Hypertension	1.009	0.990–1.029	0.371	0.304	1.009	0.991–1.027	0.324	0.474	1.006	0.987–1.025	0.566	0.644	1.005	0.990–1.021	0.500	0.443
NO hypertension	0.991	0.970–1.013	0.438		0.996	0.975–1.018	0.708		0.998	0.978–1.019	0.868		1.016	0.996–1.035	0.115	
βRBs	0.991	0.966–1.016	0.470	0.441	1.000	0.978–1.022	0.984	0.777	0.999	0.980–1.019	0.946	0.785	1.010	0.998–1.023	0.111	0.684
NO βRBs	1.019	0.992–1.047	0.162		1.018	0.991–1.045	0.191		1.014	0.989–1.041	0.279		1.009	0.984–1.034	0.480	
Statins	0.998	0.952–1.048	0.949	0.756	0.992	0.947–1.039	0.734	0.834	0.985	0.945–1.028	0.491	0.717	1.007	0.989–1.026	0.432	0.930
NO Statins	0.997	0.978–1.016	0.772		1.004	0.986–1.022	0.690		1.003	0.985–1.021	0.737		1.010	0.994–1.026	0.243	
Nitrates	0.977	0.939–1.017	0.259	0.184	0.984	0.949–1.021	0.403	0.231	0.992	0.959–1.026	0.652	0.363	0.991	0.961–1.023	0.576	0.134
NO Nitrates	1.006	0.991–1.022	0.424		1.008	0.993–1.022	0.297		1.005	0.991–1.020	0.470		1.012	1.000–1.024	0.059	
Warfarin	–	–	–	–	1.103	0.995–1.223	0.063	0.060	1.076	0.981–1.180	0.118	0.125	1.031	0.964–1.103	0.372	0.521
NO Warfarin	1.001	0.986–1.017	0.868		1.002	0.987–1.017	0.764		1.002	0.987–1.016	0.822		1.008	0.997–1.020	0.148	
Heparin	0.984	0.956–1.013	0.268	0.179	0.980	0.953–1.008	0.166	0.062	0.983	0.957–1.009	0.203	0.077	1.012	0.989–1.036	0.318	0.847
NO Heparin	1.010	0.994–1.026	0.221		1.013	0.999–1.028	0.065		1.011	0.997–1.025	0.127		1.007	0.994–1.021	0.286	
**MBP**																
CHF	1.006	0.999–1.014	0.115	0.193	1.006	0.999–1.013	0.106	0.143	1.007	1.000–1.014	0.055	0.183	1.005	0.998–1.012	0.143	0.026
NO CHF	1.017	1.007–1.027	<0.001		1.017	1.008–1.027	<0.001		1.016	1.007–1.026	<0.001		1.019	1.009–1.029	<0.001	
Valvular disease	1.010	0.996–1.024	0.152	0.867	1.010	0.997–1.023	0.133	0.884	1.013	1.001–1.025	0.041	0.762	1.008	0.997–1.020	0.150	0.614
NO valvular disease	1.010	1.004–1.017	0.002		1.010	1.004–1.017	0.002		1.010	1.003–1.017	0.002		1.011	1.005–1.018	<0.001	
Hypertension	1.010	0.996–1.024	0.168	0.669	1.011	0.997–1.025	0.090	0.647	1.009	0.995–1.022	0.201	0.961	1.010	0.999–1.024	0.080	0.517
NO hypertension	1.008	1.002–1.014	0.016		1.008	1.001–1.014	0.016		1.009	1.002–1.015	0.006		1.008	1.002–1.015	0.011	
βRBs	1.005	0.994–1.015	0.391	0.472	1.006	0.996–1.016	0.227	0.637	1.009	1.000–1.018	0.039	0.931	1.010	1.003–1.018	0.007	0.591
NO βRB	1.013	1.004–1.021	0.004		1.012	1.004–1.021	0.005		1.011	1.002–1.019	0.011		1.010	1.002–1.018	0.019	
Statins	1.021	1.005–1.036	0.010	0.099	1.014	1.000–1.027	0.045	0.235	1.015	1.002–1.029	0.027	0.150	1.017	1.004–1.030	0.010	0.087
NO Statins	1.007	1.000–1.013	0.046		1.007	1.001–1.014	0.026		1.007	1.001–1.014	0.025		1.007	1.001–1.013	0.028	
Nitrates	0.991	0.970–1.011	0.421	0.014	0.993	0.975–1.013	0.484	0.013	0.995	0.978–1.013	0.609	0.007	0.984	0.967–1.003	0.093	<0.001
NO Nitrates	1.014	1.007–1.020	<0.001		1.014	1.007–1.020	<0.001		1.014	1.007–1.021	<0.001		1.018	1.010–1.025	<0.001	
Warfarin	1.015	0.973–1.058	0.499	0.843	1.005	0.973–1.038	0.779	0.694	1.003	0.972–1.036	0.831	0.726	0.984	0.956–1.014	0.292	0.775
NO Warfarin	1.010	1.004–1.016	<0.001		1.010	1.004–1.016	<0.001		1.010	1.004–1.016	<0.001		1.011	1.005–1.017	<0.001	
Heparin	0.997	0.987–1.008	0.627	0.003	0.998	0.988–1.008	0.681	0.003	1.000	0.990–1.009	0.927	0.004	1.001	0.993–1.010	0.739	0.021
NO Heparin	1.019	1.010–1.027	<0.001		1.018	1.010–1.027	<0.001		1.018	1.010–1.027	<0.001		1.016	1.008–1.024	<0.001	
**SBP**																
CHF	0.988	0.973–1.004	0.136	0.069	0.991	0.977–1.005	0.212	0.066	0.995	0.983–1.008	0.492	0.118	1.004	0.992–1.015	0.538	0.191
NO CHF	1.010	0.998–1.021	0.098		1.009	0.998–1.020	0.118		1.008	0.997–1.019	0.138		1.010	1.001–1.019	0.026	
Valvular disease	1.016	0.993–1.039	0.172	0.230	1.014	0.994–1.033	0.166	0.211	1.009	0.990–1.027	0.361	0.616	1.010	0.996–1.024	0.179	0.878
NO valvular disease	0.998	0.988–1.008	0.674		0.999	0.989–1.008	0.774		1.002	0.992–1.011	0.740		1.007	0.999–1.015	0.087	
Hypertension	1.006	0.991–1.022	0.402	0.125	1.007	0.993–1.022	0.307	0.215	1.005	0.992–1.019	0.429	0.371	1.010	0.999–1.021	0.085	0.277
NO hypertension	0.996	0.985–1.008	0.521		0.997	0.986–1.008	0.593		1.000	0.990–1.011	0.947		1.005	0.996–1.015	0.278	
βRBs	1.006	0.993–1.018	0.389	0.086	1.007	0.996–1.019	0.218	0.108	1.010	0.999–1.021	0.062	0.051	1.013	1.003–1.022	0.007	0.056
NO βRB	0.993	0.980–1.007	0.346		0.992	0.979–1.006	0.276		0.991	0.978–1.004	0.173		0.999	0.988–1.011	0.904	
Statins	1.008	0.981–1.035	0.571	0.401	1.002	0.978–1.026	0.883	0.613	1.001	0.980–1.023	0.914	0.788	1.015	1.000–1.031	0.049	0.180
NO Statins	0.998	0.988–1.008	0.683		0.999	0.990–1.009	0.895		1.001	0.992–1.010	0.847		1.004	0.996–1.012	0.325	
Nitrates	1.001	0.981–1.021	0.918	0.977	1.002	0.984–1.020	0.859	0.911	1.002	0.985–1.018	0.848	0.814	0.993	0.978–1.008	0.364	0.019
NO Nitrates	1.002	0.991–1.012	0.750		1.002	0.992–1.012	0.758		1.003	0.993–1.012	0.548		1.012	1.004–1.020	0.003	
Warfarin	0.954	0.827–1.099	0.513	0.474	1.026	0.963–1.094	0.424	0.468	1.029	0.970–1.091	0.339	0.405	1.016	0.972–1.063	0.484	0.731
NO Warfarin	1.001	0.992–1.010	0.782		1.002	0.993–1.010	0.713		1.003	0.995–1.011	0.472		1.008	1.001–1.015	0.023	
Heparin	0.997	0.984–1.011	0.689	0.519	0.998	0.985–1.011	0.738	0.613	0.999	0.987–1.011	0.880	0.343	1.002	0.991–1.012	0.770	0.180
NO Heparin	1.002	0.989–1.015	0.806		1.002	0.989–1.014	0.781		1.004	0.993–1.015	0.489		1.009	1.000–1.019	0.055	

*Adjusted for age, gender, CHF, valvular disease, stroke, CKD, chronic bronchitis, depression, diabetes and malignant*.

*P#: P-value for interaction*.

*DBP, diastolic blood pressure; MBP, mean blood pressure; SBP, systolic blood pressure; βRB, βreceptor blockers; CHF, chronic heart failure; CKD, chronic kidney disease*.

**Table 6:** Multiple logistic regression analysis for relationship between between BP (≤55/70/110 mmHg) and mortality by stratified analysis.

**Variables**	**Hospital mortality**				**7-day mortality**				**30-day mortality**				**1-year mortality**			
	**OR**	**95% CI**	***P*** **Value**	***P#***	**OR**	**95% CI**	***P*** **Value**	***P#***	**OR**	**95% CI**	***P*** **Value**	***P#***	**OR**	**95% CI**	***P*** **Value**	***P#***
**DBP**																
CHF	0.963	0.947–0.980	<0.001	0.746	0.969	0.953–0.985	<0.001	0.755	0.969	0.953–0.985	<0.001	0.807	0.976	0.960–0.992	0.003	0.204
NO CHF	0.966	0.946–0.987	0.002		0.964	0.944–0.985	<0.001		0.965	0.946–0.986	<0.001		0.960	0.940–0.981	<0.001	
Valvular disease	0.957	0.929–0.986	0.004	0.550	0.964	0.937–0.991	0.010	0.824	0.969	0.943–0.996	0.023	0.980	0.980	0.956–1.005	0.103	0.342
NO valvular disease	0.967	0.953–0.982	<0.001		0.967	0.953–0.982	<0.001		0.967	0.953–0.981	<0.001		0.966	0.951–0.981	<0.001	
Hypertension	0.952	0.930–0.974	<0.001	0.080	0.951	0.929–0.973	<0.001	0.039	0.951	0.929–0.973	<0.001	0.026	0.943	0.920–0.968	<0.001	0.002
NO hypertension	0.972	0.956–0.989	<0.001		0.977	0.961–0.993	0.005		0.977	0.962–0.993	0.005		0.986	0.970–1.002	0.077	
βRBs	0.982	0.961–1.003	0.087	0.095	0.982	0.962–1.001	0.068	0.116	0.981	0.963–1.000	0.044	0.130	0.981	0.964–0.998	0.026	0.158
NO βRB	0.951	0.930–0.972	<0.001		0.954	0.933–0.975	<0.001		0.955	0.934–0.976	<0.001		0.961	0.940–0.983	<0.001	
Statins	0.948	0.914–0.983	0.004	0.086	0.954	0.922–0.987	0.007	0.226	0.949	0.919–0.979	<0.001	0.062	0.971	0.945–0.999	0.040	0.764
NO Statins	0.971	0.957–0.985	<0.001		0.971	0.957–0.986	<0.001		0.974	0.960–0.988	<0.001		0.974	0.960–0.989	<0.001	
Nitrates	0.989	0.945–1.035	0.625	0.102	0.992	0.949–1.037	0.726	0.075	0.987	0.948–1.028	0.532	0.103	0.977	0.945–1.009	0.161	0.233
NO Nitrates	0.961	0.947–0.975	<0.001		0.963	0.949–0.977	<0.001		0.964	0.950–0.977	<0.001		0.967	0.953–0.981	<0.001	
Warfarin	–	–	–	–	1.025	0.887–1.183	0.740	0.283	1.026	0.899–1.172	0.702	0.300	0.971	0.913–1.033	0.354	0.966
NO Warfarin	0.965	0.952–0.978	<0.001		0.966	0.953–0.978	<0.001		0.966	0.954–0.979	<0.001		0.970	0.957–0.982	<0.001	
Heparin	0.995	0.973–1.017	0.653	<0.001	0.993	0.973–1.014	0.531	<0.001	0.994	0.974–1.014	0.564	<0.001	0.997	0.977–1.016	0.731	<0.001
NO Heparin	0.947	0.931–0.965	<0.001		0.950	0.934–0.967	<0.001		0.950	0.934–0.967	<0.001		0.952	0.934–0.970	<0.001	
**MBP**																
CHF	0.985	0.961–1.009	0.227	<0.001	0.989	0.965–1.013	0.357	<0.001	0.979	0.956–1.002	0.070	0.001	0.976	0.952–1.000	0.051	0.046
NO CHF	0.913	0.877–0.950	<0.001		0.910	0.874–0.948	<0.001		0.910	0.875–0.947	<0.001		0.935	0.904–0.967	<0.001	
Valvular disease	0.984	0.944–1.025	0.440	0.176	0.987	0.948–1.027	0.513	0.134	0.985	0.949–1.022	0.417	0.051	0.988	0.955–1.022	0.487	0.052
NO valvular disease	0.951	0.928–0.975	<0.001		0.952	0.929–0.976	<0.001		0.942	0.917–0.968	<0.001		0.946	0.920–0.972	<0.001	
Hypertension	0.941	0.910–0.973	<0.001	0.079	0.941	0.910–0.974	<0.001	0.064	0.948	0.918–0.979	0.001	0.524	0.933	0.900–0.966	<0.001	0.025
NO hypertension	0.966	0.940–0.992	0.011		0.974	0.951–0.999	0.039		0.959	0.933–0.985	0.003		0.979	0.954–1.005	0.110	
βRBs	0.973	0.947–1.000	0.047	0.358	0.974	0.948–1.000	0.050	0.431	0.963	0.938–0.989	0.005	0.754	0.963	0.937–0.989	0.005	0.914
NO βRB	0.947	0.914–0.981	0.003		0.951	0.919–0.985	0.005		0.953	0.921–0.986	0.006		0.967	0.936–0.999	0.042	
Statins	0.993	0.906–1.090	0.890	0.758	0.987	0.908–1.074	0.769	0.866	0.987	0.908–1.074	0.769	0.555	0.987	0.940–1.037	0.602	0.665
NO Statins	0.965	0.945–0.985	<0.001		0.967	0.947–0.987	0.002		0.965	0.945–0.985	<0.001		0.968	0.947–0.990	0.004	
Nitrates	0.985	0.937–1.037	0.567	0.143	0.985	0.937–1.037	0.567	0.167	0.959	0.918–1.003	0.068	0.610	0.881	0.802–0.968	0.008	0.092
NO Nitrates	0.949	0.927–0.972	<0.001		0.953	0.930–0.975	<0.001		0.952	0.930–0.975	<0.001		0.966	0.944–0.988	0.003	
Warfarin	–	–	–	–	1.051	0.838–1.316	0.668	0.254	1.058	0.838–1.336	0.635	0.177	0.958	0.876–1.048	0.351	0.986
NO Warfarin	0.959	0.939–0.979	<0.001		0.960	0.940–0.979	<0.001		0.952	0.932–0.973	<0.001		0.958	0.938–0.979	<0.001	
Heparin	0.973	0.942–1.005	0.095	0.057	0.983	0.955–1.013	0.266	0.057	0.961	0.932–0.992	0.014	0.540	0.970	0.940–1.001	0.059	0.403
NO Heparin	0.944	0.918–0.971	<0.001		0.945	0.919–0.972	<0.001		0.951	0.925–0.976	<0.001		0.952	0.926–0.979	<0.001	
**SBP**																
CHF	0.982	0.974–0.990	<0.001	0.493	0.984	0.976–0.992	<0.001	0.218	0.984	0.976–0.993	<0.001	0.243	0.988	0.980–0.996	0.003	0.013
NO CHF	0.976	0.965–0.987	<0.001		0.973	0.963–0.984	<0.001		0.974	0.964–0.985	<0.001		0.967	0.955–0.980	<0.001	
Valvular disease	0.973	0.959–0.988	<0.001	0.240	0.975	0.962–0.989	<0.001	0.368	0.979	0.965–0.993	0.001	0.661	0.984	0.971–0.997	0.006	0.809
NO valvular disease	0.982	0.975–0.989	<0.001		0.982	0.975–0.989	<0.001		0.982	0.974–0.989	<0.001		0.980	0.972–0.988	<0.001	
Hypertension	0.976	0.965–0.988	<0.001	0.384	0.973	0.962–0.985	<0.001	0.161	0.973	0.962–0.985	<0.001	0.093	0.964	0.950–0.979	<0.001	0.003
NO hypertension	0.981	0.973–0.989	<0.001		0.984	0.976–0.992	<0.001		0.985	0.977–0.993	<0.001		0.989	0.981–0.998	0.012	
βRBs	0.989	0.979–1.000	0.040	0.095	0.988	0.978–0.998	0.016	0.086	0.988	0.978–0.997	0.009	0.170	0.987	0.979–0.996	0.005	0.089
NO βRB	0.969	0.957–0.982	<0.001		0.971	0.959–0.984	<0.001		0.973	0.961–0.986	<0.001		0.975	0.963–0.988	<0.001	
Statins	0.974	0.957–0.991	0.003	0.208	0.974	0.959–0.989	0.004	0.387	0.974	0.959–0.989	<0.001	0.137	0.982	0.968–0.996	0.011	0.978
NO Statins	0.982	0.975–0.989	<0.001		0.982	0.974–0.989	<0.001		0.984	0.976–0.991	<0.001		0.982	0.974–0.990	<0.001	
Nitrates	0.986	0.966–1.006	0.168	0.172	0.987	0.967–1.008	0.232	0.140	0.984	0.966–1.002	0.090	0.309	0.984	0.967–1.001	0.063	0.422
NO Nitrates	0.978	0.971–0.985	<0.001		0.978	0.971–0.985	<0.001		0.979	0.972–0.986	<0.001		0.979	0.972–0.987	<0.001	
Warfarin	–	–	–	–	1.020	0.902–1.152	0.756	0.293	1.022	0.903–1.156	0.731	0.273	0.972	0.940–1.005	0.105	0.590
NO Warfarin	0.980	0.974–0.987	<0.001		0.980	0.973–0.987	<0.001		0.981	0.974–0.987	<0.001		0.981	0.974–0.988	<0.001	
Heparin	0.991	0.981–1.001	0.093	0.301	0.991	0.981–1.001	0.083	0.349	0.992	0.982–1.002	0.100	0.121	0.994	0.984–1.004	0.211	0.600
NO Heparin	0.972	0.963–0.981	<0.001		0.973	0.964–0.982	<0.001		0.973	0.965–0.982	<0.001		0.971	0.960–0.981	<0.001	

*Adjusted for age, gender, CHF, valvular disease, stroke, CKD, chronic bronchitis, depression, diabetes and malignant*.

*P#: P-value for interaction*.

*DBP, diastolic blood pressure; MBP, mean blood pressure; SBP, arterial systolic blood pressure; βRB, βreceptor blockers; CHF, chronic heart failure; CKD, chronic kidney disease*.

## Discussion

We observed U-shaped relations between BP (SBP, MBP, and DBP) and mortality (hospital mortality, 7-day mortality, 30-day mortality, and 1-year mortality). The BP points for SBP, MBP, and DBP with the lowest mortality risk in patients with AF in our study were 110, 70, and 55 mm Hg, respectively (Figure 1). Studies on optimal BP in patients with AF have been few in the past and previous guidelines on hypertension therapy recommend tight control of BP (17–19). The clinical study including 3,947 patients with AF from the Atrial Fibrillation Follow-Up Investigation of Rhythm Management trial (AFFIRM) also suggested U-shaped curves between BP and all-cause mortality, and the risk of all-cause mortality was lowest at 140/78 mm Hg (14). In the AFFIRM study, patients with AF were either older adults or had at least one risk factor for cardiovascular events (20, 21). However, the authors further observed significantly greater mortality when patients with AF had an average BP (SBP/DBP) below 110/60 mm Hg, which is inconsistent with our finding that AF patients with SBP ≤ 110, MBP ≤ 70, or DBP ≤ 55 mm Hg tended to exhibit a reduced risk of mortality. This discrepancy may originate from different demographic characteristics and lifestyles, differences in comorbidities and treatment histories, different statistical methods, and different BP measurement methods. For example, the sample in the current study consists only of patients from the ICU. Their physiological status is worse, and there are more accompanying diseases than in ordinary patients. Previous studies have found that every 20/10 mm Hg increase in BP contributed to an increased risk of cardiovascular events in seniors with BP >115/75 mm Hg (6, 7). Roughly consistent with the findings of these previous studies, our results demonstrated that higher BP was associated with an increased risk of mortality in AF patients with SBP > 110, MBP > 70, or DBP > 55 mm Hg.

Although AF prevalence is affected by various factors, advanced age is the most important risk factor for AF (22). Existing epidemiological analyses have consistently confirmed a gradual increase in AF prevalence with advancing age (23–25). Thus, we also performed a stratified analysis by adding age as the stratification variable to evaluate the correlation between BP and mortality in patients with AF. However, our results showed that different age groups (age ≥ 65 and age <65; age ≥ 73, and age <73 years) have a little modifying effect on this relationship (data not shown). One possible explanation for these results is that the study sample consists of older adult ICU patients, and the influence of age on BP and mortality in patients with AF is disturbed by poor physiological state and accompanying diseases. Current epidemiological evidence also suggests a sex difference in the epidemiology of AF (26). A study of North American and European populations showed that the rate of AF was higher in men than women after adjustment for age. The results from the Framingham Heart Study (HFS) showed that the incidence of AF (per 1,000 person-years) was 1.6 in men and 3.8 in women (22). A significantly higher rate of AF in the male population is also observed in Asians, although there are few data (27, 28). Furthermore, one study showed that the AF prevalence was 7.4% in women and 10.3% in men among adults aged ≥65 years with Medicare beneficiaries (29). In our study, we still did not observe a modifying effect of sex, which suggests that there is no significant sex difference in this association between BP and mortality in patients with AF (data not shown). In addition to the explanation for the results described above, other potential factors need to be further studied in the future. Additionally, in AF patients with MBP >70 mm Hg or SBP >110 mm Hg, our results suggest that nitrates significantly modified the association between BP and 1-year mortality. Moreover, hypertension modified the association between DBP and mortality in patients with DBP ≤ 55 mm Hg. CHF and hypertension modified the association between MBP and mortality in AF patients with MBP ≤ 70 mm Hg and in patients with SBP ≤ 110 mm Hg, respectively. These significant results are also well explained by comorbidity and medication.

This study has several notable advantages. Our study data were obtained from the MIMIC-III database (15), which is a public critical care database that contains records from tens of thousands of ICU admissions to the Beth Israel Deaconess Medical Center from 2001 to 2012 and provides high-quality data. Professional researchers ensured the reliability and standardization of the data. Second, our study identified U-shaped associations of BP with risks of in-hospital mortality and post-hospital mortality in ICU patients with AF, providing the research evidence for controversial results on patients with AF. The SBP, MBP, and DBP levels with the lowest mortality risks in patients with AF in our study were 110, 70, and 50 mm Hg, respectively, which is inconsistent with the findings of previous relevant studies. Third, numerous disease histories and treatment histories were corrected for and stratified in our study, which improves the credibility of the conclusions of this study.

Of course, common defects in clinical research are also present in our study. Despite the MIMIC-III database prospectively providing high-quality data on ICU patients, the inevitable shortcomings of *post-hoc* analyses must be taken into consideration. Although meticulous adjustment for numerous potential confounding factors was made, regression analyses could not eliminate unknown or unmeasured variables. Overfitting models of regression analyses are likely to produce a bias toward the study hypothesis with the potential to conservatively underestimate the relationship between BP and mortality. In our results, baseline BP was recorded repeatedly, but we mainly used BP value from the first record after each patient entered the ICU ward. Therefore, the relationships of two BP measurements (maximum and minimum value BP during ICU) with mortality risk in these patients with AF were also analyzed (Supplementary Materials), suggesting a similarly U-shaped relationship, which suggested that our research results were reliable.

## Conclusions

We identified U-shaped associations between BP and in-hospital/all-cause mortality in ICU patients with AF. The BP levels with the lowest mortality risks were 110, 70, and 55 for SBP, MBP, and DBP, respectively. This study demonstrated that increased BP values when SBP >110, MBP >70, or DBP >55 mmHg are associated with a higher risk of all-cause mortality. In contrast, mortality risk declines with increasing BP when SBP ≤110, MBP ≤70, or DBP ≤55.

## Data Availability Statement

The datasets presented in this study can be found in online repositories. The names of the repository/repositories and accession number(s) can be found in the article/Supplementary Material.

## Ethics Statement

The study was conducted in accordance with the Declaration of Helsinki. The Institutional Review Boards (IRB) of BIDMC and MIT approved the project and informed consents were exempted due to all patients' data were anonymized before the data were obtained.

## Author Contributions

YS is responsible for the data analysis and writing. JH is responsible for the supervision and revision. Both authors contributed to the article and approved the submitted version.

## Conflict of Interest

The authors declare that the research was conducted in the absence of any commercial or financial relationships that could be construed as a potential conflict of interest.

## Publisher's Note

All claims expressed in this article are solely those of the authors and do not necessarily represent those of their affiliated organizations, or those of the publisher, the editors and the reviewers. Any product that may be evaluated in this article, or claim that may be made by its manufacturer, is not guaranteed or endorsed by the publisher.
