# Supplementary figures and images for: U-Shaped Association Between Blood Pressure and Mortality Risk in ICU Patients With Atrial Fibrillation: The MIMIC-III Database
Source: Front Cardiovasc Med. 2022 Jun 20;9:866260. doi: 10.3389/fcvm.2022.866260 (PMC9251341; doi:10.3389/fcvm.2022.866260)

Supplementary materials Figure 1

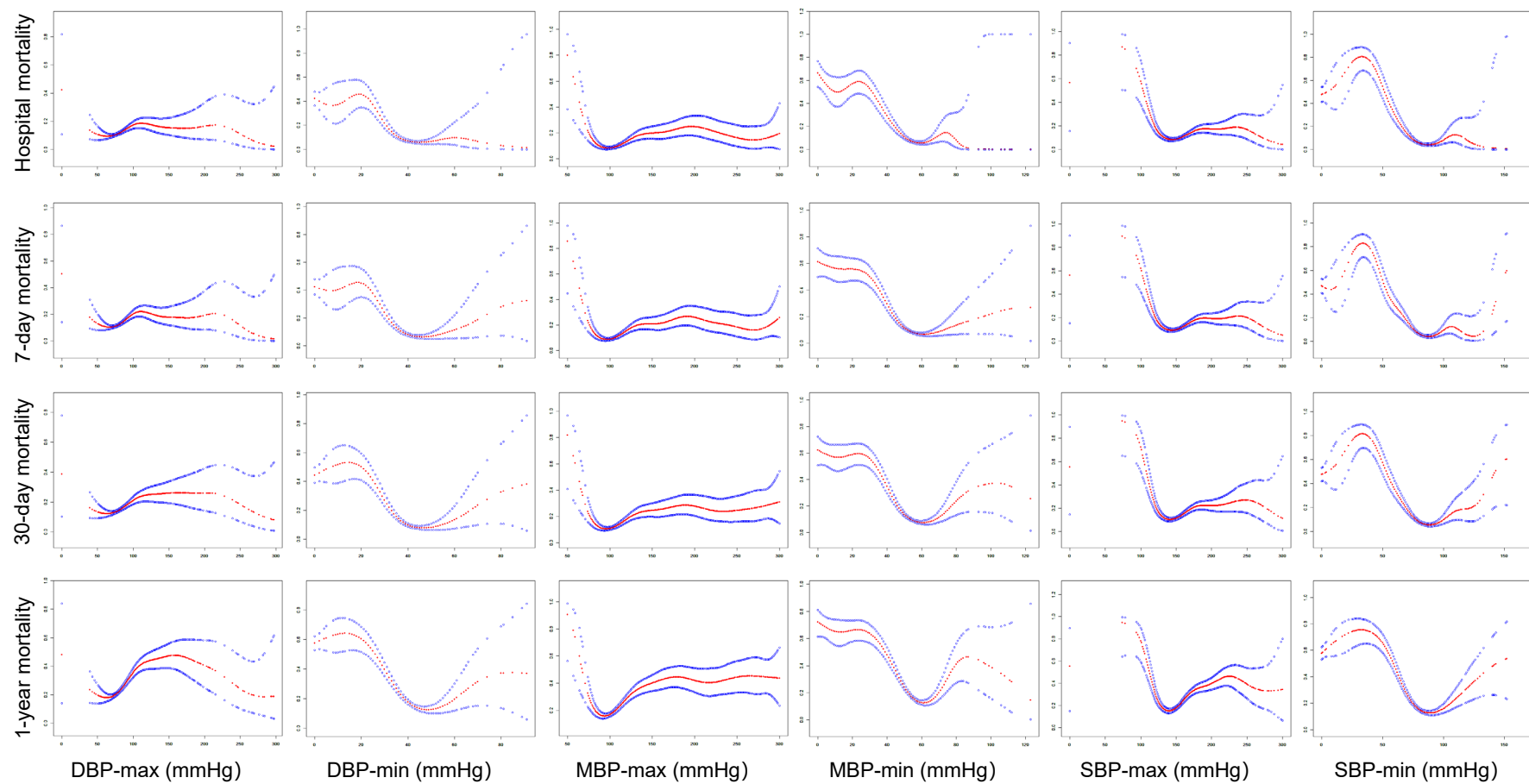

Supplement: Supplementary file 2 [file Image_1.pdf]
